# Indicator bacteria community in seawater and coastal sediment: the Persian Gulf as a case

**DOI:** 10.1186/s40201-017-0266-2

**Published:** 2017-03-10

**Authors:** Vahid Noroozi Karbasdehi, Sina Dobaradaran, Iraj Nabipour, Afshin Ostovar, Hossein Arfaeinia, Amir Vazirizadeh, Roghayeh Mirahmadi, Mozhgan Keshtkar, Fatemeh Faraji Ghasemi, Farzaneh Khalifei

**Affiliations:** 1 Department of Environmental Health Engineering, Faculty of Health, Bushehr University of Medical Sciences, Bushehr, Iran; 2 The Persian Gulf Marine Biotechnology Research Center, The Persian Gulf Biomedical Sciences Research Institute, Bushehr University of Medical Sciences, Boostan 19 Alley, Imam Khomeini Street, Bushehr, Iran; 3 Systems Environmental Health, Oil, Gas and Energy Research Center, The Persian Gulf Biomedical Sciences Research Institute, Bushehr University of Medical Sciences, Bushehr, Iran; 4 The Persian Gulf Tropical Medicine Research Center, The Persian Gulf Biomedical Sciences Research Institute, Bushehr University of Medical Sciences, Bushehr, Iran; 5 Environmental Health Department, School of Public Health, Iran University of Medical Sciences, Tehran, Iran; 6 The Persian Gulf Studies and Researches Center Marine Biotechnology Department, Persian Gulf University, Bushehr, Iran

**Keywords:** Bushehr coastal, Indicator bacteria, Persian Gulf, Sediment texture

## Abstract

**Background:**

The aim of present work was to assess the concentration levels as well as vertical distribution of indicator bacteria including total coliform, fecal coliform, Pseudomonas aeruginosa, and Heterotrophic Plate Count (HPC) in the marine environment (seawater and coastal sediments) and evaluate the correlation between indicator bacteria and some physicochemical parameters of surface sediments as well as seawaters.

**Methods:**

A total number of 48 seawater and sediment samples were taken from 8 stations (each site 6 times with an interval time of 2 weeks) between June and September 2014. Seawater and sediment samples were collected from 30 cm under the surface samples and different sediment depths (0, 4, 7, 10, 15, and 20 cm) respectively, along the Persian Gulf in Bushehr coastal areas.

**Results:**

Based on the results, the average numbers of bacterial indicators including total coliform, fecal coliform, and *Pseudomonas aeruginosa* as well as HPC in seawater samples were 1238.13, 150.87, 8.22 MPN/100 ml and 1742.91 CFU/ml, respectively, and in sediment samples at different depths (from 0-20 cm) varied between 25 × 10^3^ to 51.67 × 10^3^, 5.63 × 10^3^ to 12.46 × 10^3^, 17.33 to 65 MPN/100 ml, 36 × 10^3^ to 147.5 × 10^3^ CFU/ml, respectively. There were no statistically significant relationships between the indicator organism concentration levels with temperature as well as pH value of seawater. A reverse correlation was found between the level of indicator bacteria and salinity of seawater samples. Also results revealed that the sediment texture influenced abundance of indicators bacteria in sediments. As the concentration levels of indicators bacteria were higher in muddy sediments compare with sandy ones.

**Conclusion:**

Result conducted Bushehr coastal sediments constitute a reservoir of indicator bacteria, therefore, whole of the indicators determined were distinguished to be present in higher levels in sediments than in the overlying seawater. It was concluded that the concentration levels of microbial indicators decreased with depth in sediments. Except total coliform, the numbers of other bacteria including fecal coliform, *Pseudomonas aeruginosa* and HPC bacteria significantly declined in the depth between 10 and 15 cm.

## Background

Recreational water and beaches are often considered as a place where sensitive individuals may contact with microbial contaminations [1]. These areas are susceptible to fecal contamination from wastewater, septic leachate, farming drainage, livestock and domestic animals, or nonpoint sources of human and animal waste [2]. Fecal contamination in maritime areas can be dangerous to recreational users because feces may contain pathogenic microorganisms that can be ingested and bring intestinal problem [3]. Epidemiological surveys have revealed the positive relationship between fecal contamination at marine beaches and swimming-related diseases [4]. Microbial indicators have been utilized worldwide to show if a water body is contaminated by fecal contamination. Some of these indicators, i.e. fecal coliforms, *E. coli* and *Enterococcus spp*., are used to monitor the fecal contamination of seawater bodies worldwide [5]. Microbial impairment of drinking, irrigation, or recreational seawaters is generally monitored using concentration levels of fecal indicator bacteria [6]. But other bacteria including *Pseudomonas aeruginosa*, a gram-negative opportunistic human pathogen, and HPC bacteria may also be useful in defining seawater body quality [7]. Exceeding contents of indicator bacteria in seawater and sediments have been related to increased risk of pathogenic microorganism-induced sickness to humans [8]. Various researches have documented an elevated risk of contracting gastrointestinal diseases, skin infections as well as acute respiratory infections after exposure with recreational waters and seawater body with increased concentrations of indicator bacteria [9–12].

**Fig. 1:**
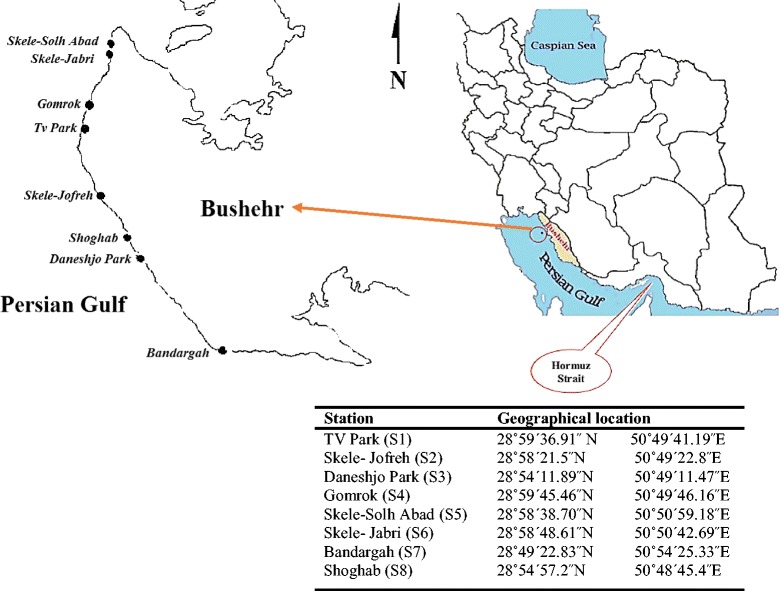
Locations of sampling stations in the study area

Within aquatic systems, it is highlighted that the indicator microorganisms can be highly related to the sediment fraction [13, 14]. This relationship is due to four ecological performances of sediments; 1) provision a place for microbial attachment [15] serves as a favorable organic substance and nutrients for microbes [16], 3) protection from environmental stresses such as sunlight UV [17], protozoan grazing [18], etc., and 4) extracellular polymeric substances (EPS) of bacteria, which enhance sediment flocculation by coagulating and attaching particles together to create a floc matrix and in turn results in an increased downward flux of sediment [19] and accordingly connected bacteria (with potential pathogens) to the sediment [20]. In general, indicator bacteria can stay alive much longer in sediment than in the water column in both freshwater and maritime environments and many studies have confirmed this [21–24]. Pachepsky and Shelton [25] and Brinkmeyer et al. [26] observed significant correlations between fecal indicator bacteria in the seawater column and underlying sediments. They found that the levels of indicator bacteria in sediments considerably higher than seawaters. Koirala et al. also found that numbers of indicator organisms in sediments are greater than in water samples due to protection behavior of sediments [17]. There are some activities related to sediment resuspension in coastal areas such as commercial or recreational boating and storms that can lead to considerable effects on microbial loads of water [27]. In addition, recreational activity and wave action in the swash zone of the coast can also contribute to re-suspension the bacteria from the sediment and consequently may predispose human to health risk [1]. Bushehr province with a long coastline (more than 707 kilometers) along the Persian Gulf and its strategic and geopolitical position, as one of the most important port, is located in southwestern Iran and northern part of the Persian Gulf (Fig. 1). Bushehr as energy capital in Iran is facing with industrial pollution in its marine environment [28] and its region is of special interest for environmental studies [29–33]. The climate is warm and wet in summer and mild in winter. Swimming in the Persian Gulf and playing in the coastline areas are the most important entertainments of people in the Bushehr port. Also, there are sporadic studies on organism’s concentrations in the coastal areas of the Persian Gulf but to our best knowledge there is no report yet on comprehensive and baseline data on indicator organisms profile in seawaters and sediments along the Persian Gulf. In this work for the first time in the region of the Persian Gulf, we aimed to (1) assess the concentration levels of indicator bacteria in different depths of surface sediments and seawaters as baseline information in the region (2) mapping and kriging interpolations of the microbial contamination in the surface sediments and seawaters (3) ascertain the correlation between indicator bacteria and some physicochemical parameters of surface sediments as well as seawaters.

## Methods

### Chemicals and reagents

The employed media including Lactose broth, Brilliant green, EC broth and R_2_A agar media were prepared from Merck, Germany. The Asparagine broth and Acetamide broth media were prepared from Sigma-Aldrich, USA.

### Study area and sediment sample collection

Seawater and sediment sampling were done between June and September 2014. A total number of 48 samples were taken from 8 sampling sites, including TV Park (S1), Skele-Jofreh (S2), Daneshjo Park (S3), Gomrok (S4), Skele-Solh Abad (S5), Skele-Jabri (S6), Bandargah (S7), and Shoghab (S8) (each site 6 times with an interval time of 2 weeks), which were located in the intertidal zone along the Persian Gulf in Bushehr beach zone (Fig. 1). First, seawater samples were collected from 30 cm under the surface (to avoid direct effect of sun ultraviolet radiation on the water surface layer) using a sterile 500 mL glass vessel (for bacterial analyses) and an open-mouthed bottle (for physicochemical parameters analysis). Next, surface sediments were collected from different depth (including 0, 4, 7, 10, 15 and 20 cm) using an Ekman steel grab sampler (25 × 25 × 25 cm^3^). For each sampling sites, 2–4 sampling points were selected based on the place or shape, and samplings was carried out. The pH of each samples were measured in the place directly after sampling using a U-50 multi-parameter water quality checker (HORIBA, Germany). Samples were placed in a cold box (temperature roughly 4 ˚C and darkness) [34] and directly transported to the lab in coolers on ice within 2 hours.

### Media and procedures for bacterial analysis

All microbial indicator analyses including total coliform, fecal coliform, *Pseudomonas aeruginosa* as well as HPC bacteria were done according to standard methods [34]. Lactose broth, EC broth and asparagine broth were employed to determine the most probable number (MPN) per 100 ml of total coliforms, fecal coliforms, as well as *Pseudomonas aeruginosa* respectively, using a five-tube multiple-dilution technique. R_2_A agar was used to ascertain the colony forming unit (CFU) per ml of HPC bacteria, using the spread plate technique. In the case of sediment samples, sediments were mixed thoroughly and diluted 1:10 with sterile distilled water (1 g of sediment added into 9 ml of sterile distilled water). This mixture was centrifuged with a speed of 8000 rpm for l-2 min and then was left to stand for 5-10 min to allow big particles to settle. Sediment suspensions were subsequently processed by the similar procedures as for water samples.

### Grain size analysis of sediment samples

Sediment samples were collected by a grab sampler and coning and quartering technique was used to prepare sediments for grain size analysis [35]. Coning and quartering method involves five steps including: (1) pour the samples onto a flat surface to form a cone (2) flatting the cone (3) divide cone in half (4) divides halves into quarters and discard alternate quarters (5) two quarters are retain and mix together, reform cone and repeats steps until remaining sample be in a correct amount for analysis). After 5 cited steps, sediment sample was kept in a polythene bag labeled with number and location and transferred to the laboratory by cold box and stored in the freezer at -20 °C until grain size examination according to Buchanan’s method [36]. For analysis, sediment dried for 24 hours at 70 °C in Heraeus oven (UT 6420 model). 25 grams of dried sediment of each sample were put in a flask containing 250 ml of distilled water. Then 10 ml of 2.6 grams per liter of sodium hexametaphosphate [Na (PO_3_)_6_] solution was added to the flask contents. After stirring the solution three times, each time for nearly 15 minutes, it was kept in the laboratory for 24 hours. In order to dry, the solution was placed in chines plates and then moved to the oven at 70 °C for 24 hours. After drying , samples were sieved by shaker Heraeus device (Analysette 3PRO model), and a series of sieves including 4, 2, 1, 0.5, 0.25, 0.125 and 0.0625 mm which climbed on each other, respectively and a container were placed under them (for weight the particles smaller than 0.0625 mm). Each sample was kept on device for 15 minutes. After that the sediment remaining on each sieve, and sediments of the lower container, weighed carefully with an accuracy of 0.1 mg. By multiplying the weight of each sieve in 4, the percent of its grain size was obtained. Finally as a percentage of dry matter in the sediment, have been reported in 4 different ranges (coarse sand (>500 μm), medium sand (500-250 μm), fine sand (250-125 μm), mud (<125 μm)). So the dominant group determined the types of grain size.

### Data analysis

Statistical processing of data was done by using the SPSS version 20 (IBM Corp., USA). The normality of data was checked by the Shapiro-Wilk test before analyzing. Descriptive statistics were applied for presentation of total coliform, fecal coliform, *Pseudomonas aeruginosa* as well as HPC concentration levels in seawater and sediment samples. Parametric Pearson test was applied to establish correlations between association of sediment and seawater characteristics and concentration levels of indicator bacteria. ArcMap 10.2 Geographical Information System (GIS) (ESRI, Redlands, CA) was also used as an appropriate tool for mapping and kriging interpolations of the bacterial concentration levels.

## Result and discussion

### Seawater

#### Number of bacterial indicators

**Fig. 2:**
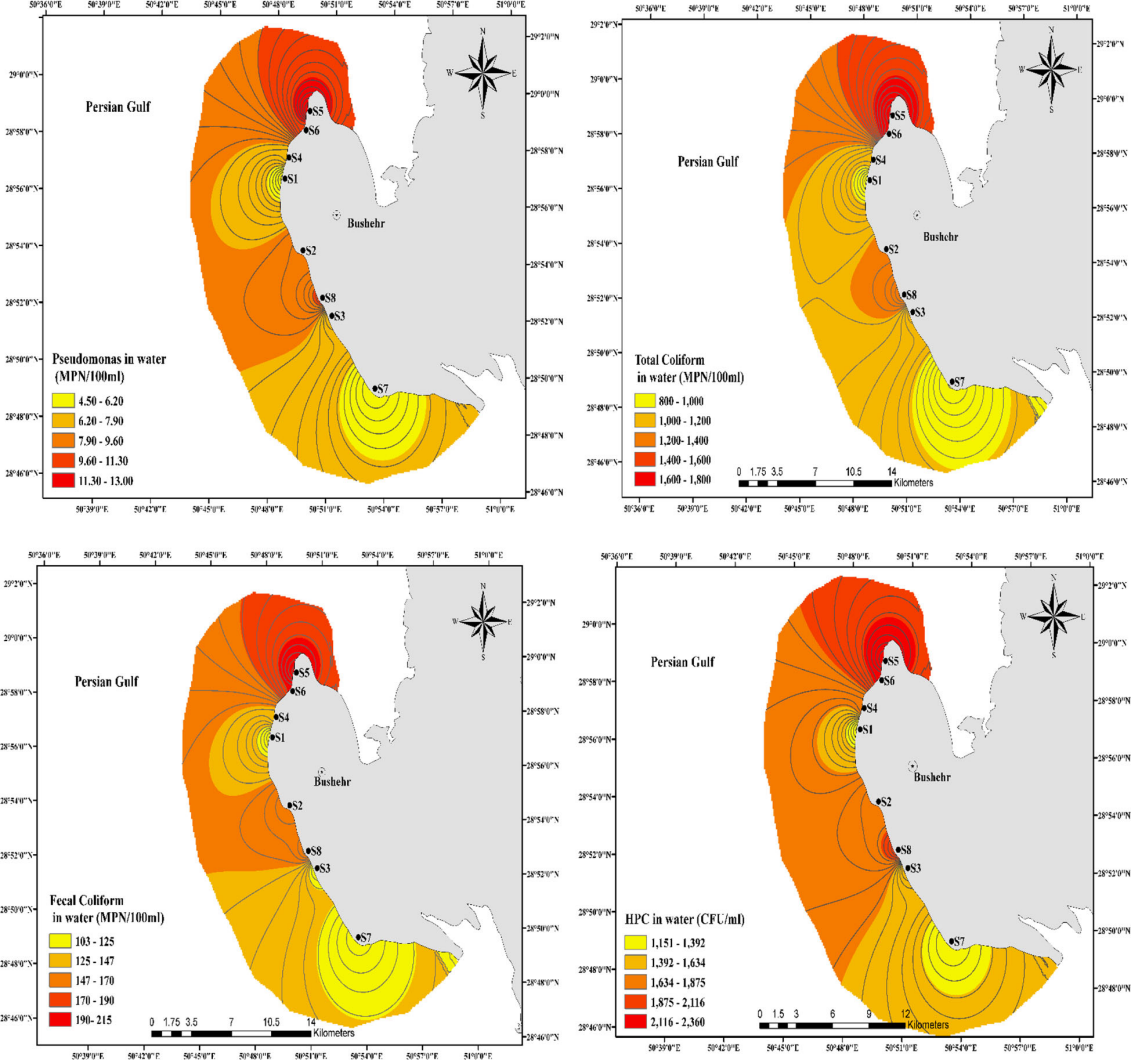
The spatial distribution of bacterial organisms in seawater samples of Bushehr coastal areas

The concentration levels of bacterial indicators in seawater samples of Bushehr coastal areas are presented in Fig. 2 and related data is summarized in Table 1. The average numbers of bacterial indicators including total coliform, fecal coliform, and *Pseudomonas aeruginosa* as well as HPC in seawater samples were 1238.13, 150.87, 8.22 MPN/100 ml and 1742.91 CFU/ml, respectively.

**Table 1:** The mean, SD, minimum and maximum values of bacterial indicator levels in seawater samples at different stations (maximum values are expressed as bold italics; minimum values as bold underlined)

Indicator bacteria	Station	N	Mean	Std. Deviation	Minimum	Maximum
Total coliform (MPN/100 ml)	1	6	**800**	112.07	600	920
	2	6	1220	184.93	920	1400
	3	6	1000	161.12	680	1100
	4	6	1050	168.99	750	1200
	5	6	***1800***	328.00	1200	2200
	6	6	1750	339.76	1100	2000
	7	6	885	111.23	660	950
	8	6	1400	247.54	920	1600
Average		48	1238.12	206.7	853.75	1421.25
Fecal coliform (MPN/100 ml)	1	6	**103.67**	15.57	79	120
	2	6	165	8.07	160	180
	3	6	111.17	17.11	94	140
	4	6	143.33	20.15	110	170
	5	6	***213.33***	72.67	110	330
	6	6	193.33	43.29	140	260
	7	6	107.17	16.29	79	130
	8	6	170	38.14	110	230
Average		48	150.875	28.91125	110.25	195
Pseudomonas (MPN/100 ml)	1	6	**4.50**	1.22	3	6
	2	6	8.50	2.07	5	11
	3	6	6.33	2.16	3	9
	4	6	7.33	2.58	4	11
	5	6	***13.00***	2.28	11	17
	6	6	11.00	1.414	9	13
	7	6	4.83	1.169	3	6
	8	6	10.33	1.966	7	12
Average		48	8.227	1.857	5.625	10.625
HPC (CFU/ml)	1	6	**1150.00**	89.443	1000	1250
	2	6	1841.67	724.166	1100	2800
	3	6	1350.00	420.095	940	1850
	4	6	1743.33	406.776	1350	2500
	5	6	***2358.33***	941.497	1500	3500
	6	6	2173.33	344.770	1890	2700
	7	6	1248.33	217.754	970	1610
	8	6	2078.33	302.815	1640	2400
Average		48	1742.91	430.914	1298.75	2326.25

The highest average numbers of bacterial indicators in seawater samples between all stations were found in S5 and S6, which were the samples collected from the Solh-Abad and Jabari fishing ports with more frequent anthropogenic activities. Hamilton et al. have previously reported that pollutants from anthropogenic -influenced sources may carry diverse bacteria into the beaches and seawaters [37]. These cited fishing ports were also close to the pisciculture and aquaculture zones for fish and shellfish. Fish and shellfish are effective filter feeders and concentrate high levels of aquatic microorganisms such as fecal coliform and other bacteria in their bodies and may be built up to contents serious to human health. Most incidents of fecal contamination of aquaculture areas are ascribed to anthropogenic origins of such as illegal discharges from boats, inadequately maintained septic systems and run-off from farms [36]. Due to such risks to human health, it is proposed that all fishing ports with high concentration levels of indicators bacteria (like these two stations in Bushehr coastal area) rigidly monitored and categorized by individual state health authorities. Generally, ports ‘approved’ for fishing must not exceed particular amounts of contamination, especially threshold values of fecal coliforms.

In addition to above mentioned two stations, one of the highest average numbers of bacterial indicators was found in S8 with an average levels of 1400, 170, 10.33 MPN/100 ml and 2078.33 CFU/ml for total coliform, fecal coliform, *Pseudomonas aeruginosa*, as well as HPC respectively. This station is located in coastal area of the Shoghab Park, which is facing wide variety anthropogenic sources such as swimmers feces, throwing up garbage via tourisms, domestic wastewater treatment and disposal practices that may lead to the entrance of high levels of coliform bacteria and enteric human pathogens into the seawater. This area is also frequently utilized for recreational applications such a swimming, recreational fishing and recreational boating. Fecal pollution at swimming marine area can be dangerous to human health because feces may comprise bacteria, viruses and protozoa and there is possibility of feeding by swimmers which leads to various diseases [1].

### Physical and chemical factors

**Fig. 3:**
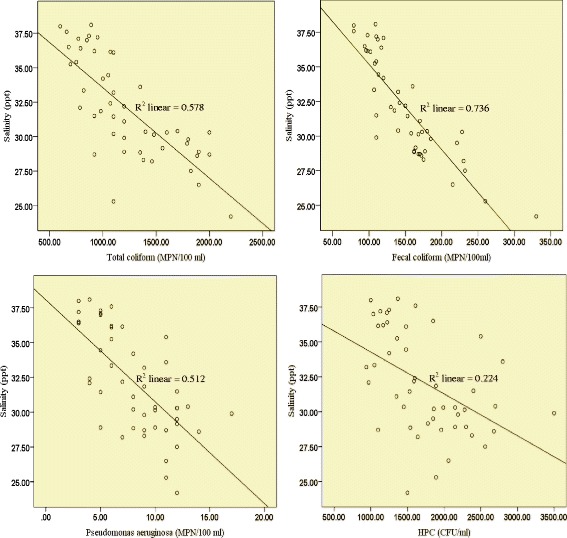
Pearson correlation between the levels of all examined indicator organisms and salinity of seawater samples

**Fig. 4:**
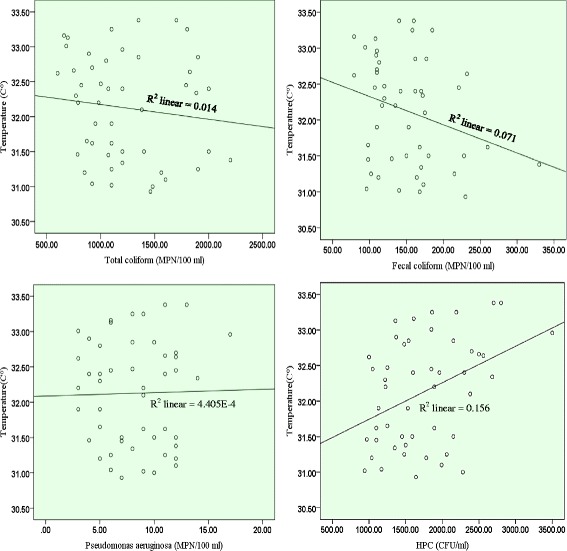
Pearson correlation between the concentrations of all examined indicator bacteria and temperature of seawater samples

**Fig. 5:**
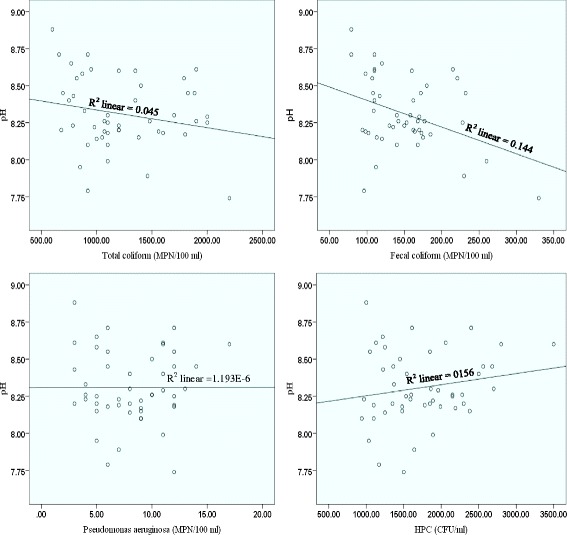
Pearson correlation between the concentrations of all examined indicator bacteria and pH of seawater samples

The physical and chemical characteristics of seawater samples from the eight stations were determined and the maximum, minimum, mean and standard deviation values are presented in Table 2. The results of physical parameter measurements in seawater samples showed that these parameters are not tangible changed at different stations. The mean values of temperature and pH in seawater samples were in the range of 31.5-32.5°C and 8.16-8.41 respectively. The salinity in seawater samples was in the range between 27.7 and 37 ppt. Pearson analysis showed that there was a significant inverse correlation between the concentrations levels of total coliforms, fecal coliforms, and *Pseudomonas aeruginosa* and salinity of seawater samples, and a weak inverse correlation between salinity and HPC bacteria (Fig. 3). Therefore, the data revealed that with decreasing salinity, there was a corresponding increase in the values of indicator bacteria in seawater samples. A negative effect of salinity is attributed to specific characteristics of sea water, such as osmotic pressure and the toxicity of inorganic salts [38]. Most studies showed a negative correlation between values of indicator bacteria and salinity. For example, Rozen and Belkini found a reverse correlation between the level of *E. coli* and water salinity in seawater [39]. An inverse correlation between survival of *E. coli* and salinity of water has also been demonstrated by Anderson et al. [40]. In contrast to our studies, Jozić et al showed that there was no statistically significant effect of salinity on the *E. coli* bacteria [41]. Also Pearson analysis showed there were no statistically significant correlations between temperature and pH parameters and indicator organisms (Fig. 4 and Fig. 5). Similarly, Guyal et al. found that the indicator organisms (total coliforms, fecal coliforms, and salmonellae) were no statistically significant relationships with temperature, pH, turbidity, and suspended solids contents of seawater [42].

**Table 2:** The mean, SD, minimum and maximum values of temperature, pH and salinity in seawater samples at various stations (maximum values are expressed as bold italics; minimum values as bold underlined)

	Station	N	Mean	Std. Deviation	Minimum	Maximum
Temperature (°C)	1	6	31.83	0.637	31.04	32.62
	2	6	32.00	0.515	31.34	32.66
	3	6	32.00	0.860	31.02	33.01
	4	6	32.48	0.805	31.62	33.38
	5	6	32.17	0.696	31.38	32.96
	6	6	31.83	0.422	31.04	32.62
	7	6	***32.50***	0.698	31.46	33.16
	8	6	**31.50**	0.724	30.93	32.07
Average		48	32.04	0.67	31.23	32.81
pH	1	6	8.38	0.423	7.79	8.88
	2	6	8.35	0.187	8.10	8.60
	3	6	**8.16**	0.037	8.10	8.20
	4	6	8.26	0.071	8.20	8.40
	5	6	8.41	0.330	7.74	8.60
	6	6	8.21	0.117	7.99	8.30
	7	6	***8.48***	0.179	8.23	8.71
	8	6	8.23	0.267	7.89	8.71
Average		48	8.227	0.201	8.01	8.55
Salinity (ppt)	1	6	***37***	0.648	36.2	38
	2	6	30.1	1.859	28.7	33.6
	3	6	35.1	1.334	33.2	36.5
	4	6	32.4	1.545	31.1	35.4
	5	6	**27.7**	2.126	24.2	29.9
	6	6	28.9	1.898	25.3	30.4
	7	6	35.6	2.459	32.1	37.6
	8	6	29.6	1.283	28.2	31.5
Average		48	32.05	1.644	29.875	34.112

In another study, Shibata et al. reported that except total coliform, there were no statistically significant relationships between the concentrations levels of enterococci, *Escherichia coli*, fecal coliform, and *C. perfringens* and physical–chemical parameters (rainfall, temperature, pH, and salinity) [43]. But Blaustein et al. [44] and Sampson et al. [45] found that temperature was a major factor in the survival of *E. coli* in surface waters. In another study Placha et al. reported that the survival of *Salmonella typhimurium* and indicator bacteria (coliform and faecal coliform bacteria and faecal streptococci) was considerably affected by temperature [46].

### Sediment

#### Number of bacterial indicators

**Fig. 6:**
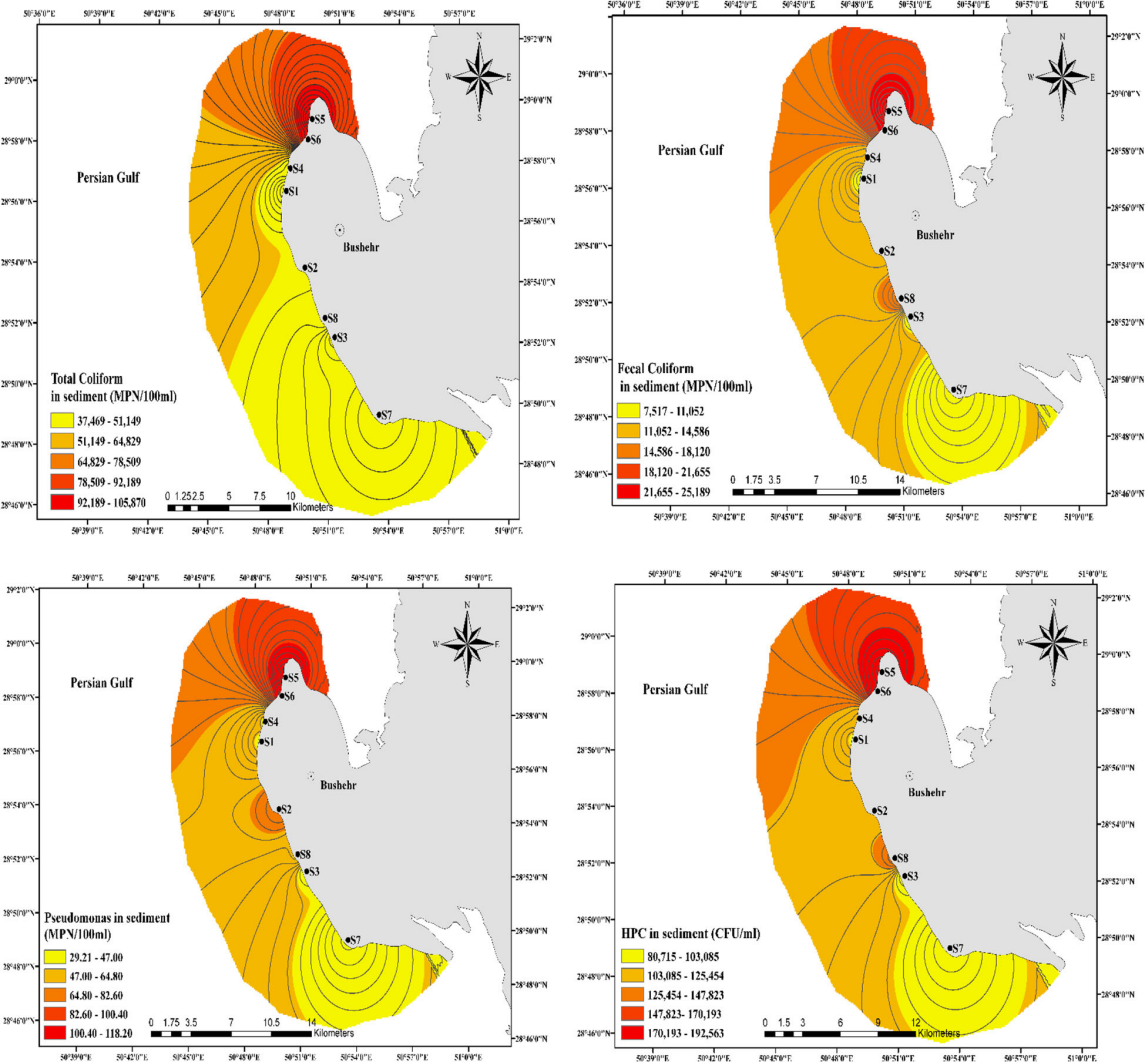
The spatial distribution of bacterial organisms in sediment samples of Bushehr coastal areas

**Fig. 7:**
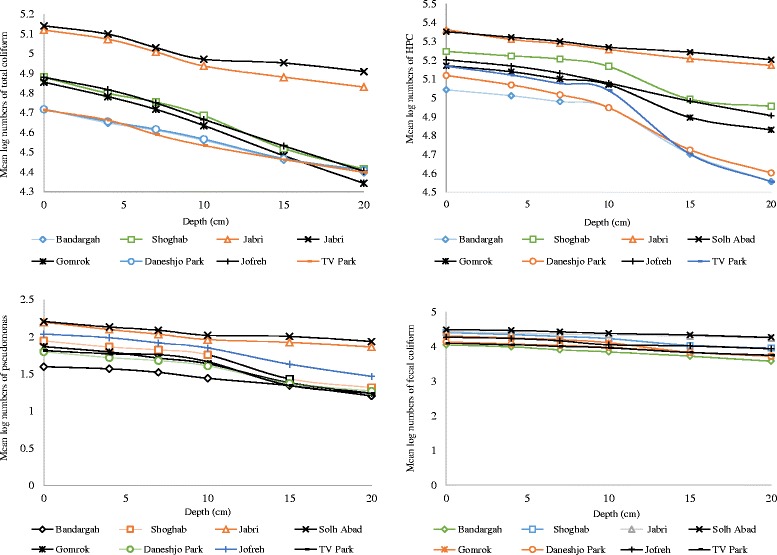
Compare the mean log values of total coliform, fecal coliform, pseudomonas aeruginosa and HPC bacteria between eight stations at various depths

The concentration levels of bacterial indicators in sediment samples of Bushehr coastal are given in Fig. 6 and associated data is summarized in Table 3. The mean log values of bacterial indicators between eight stations at various depths were compared and are shown in Fig. 7.

**Table 3:** The mean, SD, minimum and maximum values of bacterial indicators levels in various stations at different depths of sediment (maximum values are expressed as bold italics; minimum values as bold underlined)

	Depth (cm)	Station 1	Station 2	Station 3	Station 4	Station 5	Station 6	Station 7	Station 8
Total coliform (MPN/100 ml)	0	***51.67 × 10*** ^***3***^	***75.83 × 10*** ^***3***^	***52.17 × 10*** ^***3***^	***71.67 × 10*** ^***3***^	***138.33 × 10*** ^***3***^	***131.67 × 10*** ^***3***^	***52.17 × 10*** ^***3***^	***75.83 × 10*** ^***3***^
	4	46 × 10^3^	65.66 × 10^3^	45.33 × 10^3^	60.5 × 10^3^	125.67 × 10^3^	118.33 × 10^3^	44.5 × 10^3^	62.83 × 10^3^
	7	38.83 × 10^3^	56.17 × 10^3^	41.33 × 10^3^	52.16 × 10^3^	107.17 × 10^3^	102 × 10^3^	40.83 × 10^3^	56.83 × 10^3^
	10	34.17 × 10^3^	46.33 × 10^3^	36.83 × 10^3^	43.17 × 10^3^	93.5 × 10^3^	86.5 × 10^3^	36.33 × 10^3^	48.5 × 10^3^
	15	29 × 10^3^	34 × 10^3^	29.33 × 10^3^	30.33 × 10^3^	89.66 × 10^3^	76 × 10^3^	29 × 10^3^	33 × 10^3^
	20	**25 × 10** ^**3**^	**25.33 × 10** ^**3**^	**25.67 × 10** ^**3**^	**22 × 10** ^**3**^	**81 × 10** ^**3**^	**67.66 × 10** ^**3**^	**25 × 10** ^**3**^	**26 × 10** ^**3**^
	Average	37.44 × 10^3^	50.55 × 10^3^	38.44 × 10^3^	46.63 × 10^3^	105.88 × 10^3^	97.02 × 10^3^	37.97 × 10^3^	50.5 × 10^3^
Fecal coliform (MPN/100 ml)	0	***12.46 × 10*** ^***3***^	***18.66 × 10*** ^***3***^	***13.73 × 10*** ^***3***^	***20.16 × 10*** ^***3***^	***31 × 10*** ^***3***^	***27.17 × 10*** ^***3***^	***11.23 × 10*** ^***3***^	***25.66 × 10*** ^***3***^
	4	11.33 × 10^3^	17.16 × 10^3^	11.9 × 10^3^	17.66 × 10^3^	29.33 × 10^3^	24.66 × 10^3^	9.8 × 10^3^	22.33 × 10^3^
	7	10.21 × 10^3^	14.66 × 10^3^	10.8 × 10^3^	15.73 × 10^3^	26.83 × 10^3^	23 × 10^3^	8.2 × 10^3^	19.67 × 10^3^
	10	9.26 × 10^3^	11.33 × 10^3^	9.56 × 10^3^	12.86 × 10^3^	24 × 10^3^	21.67 × 10^3^	7.2 × 10^3^	17.7 × 10^3^
	15	6.76	10.5 × 10^3^	6.83 × 10^3^	6.83 × 10^3^	21.67 × 10^3^	20 × 10^3^	5.26 × 10^3^	10.8 × 10^3^
	20	**5.63 × 10** ^**3**^	**8.66 × 10** ^**3**^	**5.26 × 10** ^**3**^	**5.26 × 10** ^**3**^	**18.33 × 10** ^**3**^	**17.33 × 10** ^**3**^	**3.76 × 10** ^**3**^	**9 × 10** ^**3**^
	Average	9.28 × 10^3^	13.5 × 10^3^	9.68 × 10^3^	13.09 × 10^3^	25.19 × 10^3^	22.3 × 10^3^	7.52 × 10^3^	17.42 × 10^3^
Pseudomonas (MPN/100 ml)	0	***65***	***108.83***	***62.6***	***73.5***	***160***	***156.6***	***39.5***	***87.6***
	4	58.83	97	52.3	62.3	135	125	37	72.6
	7	57.33	82.6	47.6	51.5	122.3	108	33.16	66.3
	10	46.1	70.8	40.5	43.3	104.5	91.6	27.6	57.16
	15	22	42.6	23.66	24	101	84	22	27
	20	**17.33**	**29.3**	**18.6**	**17.33**	**86.33**	**73**	**16**	**20. 6**
	Average	44.43	71.85	40.88	45.32	118.19	106.36	29.21	62.13
HPC (CFU/ml)	0	***147.5 × 10*** ^***3***^	***159.42 × 10*** ^***3***^	***131.67 × 10*** ^***3***^	***148.35 × 10*** ^***3***^	***224.83 × 10*** ^***3***^	***230.66 × 10*** ^***3***^	***110.5 × 10*** ^***3***^	***176.83 × 10*** ^***3***^
	4	132.5 × 10^3^	148 × 10^3^	117.33 × 10^3^	138 × 10^3^	210.17 × 10^3^	204.67 × 10^3^	102.8 × 10^3^	167.33 × 10^3^
	7	119.42 × 10^3^	135.5 × 10^3^	104.17 × 10^3^	126.17 × 10^3^	199.83 × 10^3^	194.5 × 10^3^	95.62 × 10^3^	161.17 × 10^3^
	10	109.63 × 10^3^	119.92 × 10^3^	88.83 × 10^3^	117.83 × 10^3^	186 × 10^3^	180.67 × 10^3^	89.04 × 10^3^	147.17 × 10^3^
	15	50.33 × 10^3^	96.33 × 10^3^	53 × 10^3^	78.66 × 10^3^	175 × 10^3^	161.67 × 10^3^	50.33 × 10^3^	98.33 × 10^3^
	20	**36 × 10** ^**3**^	**80.67 × 10** ^**3**^	**40 × 10** ^**3**^	**67.66 × 10** ^**3**^	**159.67 × 10** ^**3**^	**149 × 10** ^**3**^	**36 × 10** ^**3**^	**90.33 × 10** ^**3**^
	Average	99.23 × 10^3^	123.3 × 10^3^	89.17 × 10^3^	112.78 × 10^3^	192.58 × 10^3^	186.86 × 10^3^	80.71 × 10^3^	140.19 × 10^3^

As seen in Table 3, the levels of total coliforms, fecal coliform, *pseudomonas aeruginosa* as well as HPC in sediment samples at different depths (from 0-20 cm) in stations (1 to 8) varied between 25 × 10^3^ and 51.67 × 10^3^, 5.63 × 10^3^ and 12.46 × 10^3^, 17.33 and 65, 36 × 10^3^ and 147.5 × 10^3^ respectively. The indicators bacteria counts at S5 and S6 sites, (hereinafter termed as the polluted sites) were considerably greater than those at the other six sites. It is also possible that the types of nutrients present in two stations are different from other stations and are more easily utilized by indicators bacteria. The ecological occurrence of indicators organisms in coastal area sediment has been documented [1, 43, 47]. In our study like many former studies [14, 23, 40], the numbers of indicator organisms in all examined sampling sites were higher for sediment samples compare to seawater samples. This may be due to the fact that sediments probably serve as a suitable environment for bacterial survival [48]. In present work, indicator bacteria were 10 to 100 times higher in sediments than in seawater samples. Crabill et al. reported that the average counts of fecal coliforms in sediment samples were 2200 times higher than the water counts [49]. In another research by Davies and Bavor, they confirmed that sediments may have 10 to 10,000 times higher amounts of fecal indicator organisms compare with the overlying seawater [22]. This may be due to the adsorption and sedimentation tend to remove organisms from suspension and concentrate them in bottom sediments [39]. But many studies reviewed by Pachepsky and Shelton [25], Brinkmeyer et al. [26] reported no correlations between indicators bacteria in the water column and underlying sediments.

### The general decay of indicators number in sediment

Our results showed that the concentration levels of bacterial indicators decreased with depth (Table 4). This may be due to the death and inactivation of bacteria with depth. Because sediments is a natural filter that ensnares environmental particulates, organic substance and microorganisms [50, 51]. Our study are in accordance with former studies reported by Brinkmeyer et al. [26], Alm et al. [1], Haller et al. [21], and Pachepsky and Shelton [25].

**Table 4:** Mean concentration levels and mean log numbers of indicators bacteria (the average of eight stations) in sediment samples at various depths

Depth (cm)		N	Mean	Log number	Std. Deviation	Minimum	Maximum
Total coliform (MPN/100 ml)	0	48	81.17 × 10^3^	4.909	34.65 × 10^3^	43 × 10^3^	180 × 10^3^
	4	48	71.34 × 10^3^	4.853	32.58 × 10^3^	34 × 10^3^	170 × 10^3^
	7	48	61.92 × 10^3^	4.792	27.54 × 10^3^	33 × 10^3^	140 × 10^3^
	10	48	53.17 × 10^3^	4.725	23.99 × 10^3^	31 × 10^3^	130 × 10^3^
	15	48	43.78 × 10^3^	4.641	25.32 × 10^3^	26 × 10^3^	120 × 10^3^
	20	48	37.21 × 10^3^	4.571	23.76 × 10^3^	21 × 10^3^	110 × 10^3^
Fecal coliform (MPN/100 ml)	0	48	20 × 10^3^	4.301	8.17 × 10^3^	9 × 10^3^	35 × 10^3^
	4	48	18 × 10^3^	4.255	7.68 × 10^3^	7.9 × 10^3^	33 × 10^3^
	7	48	16.1 × 10^3^	4.207	7.189 × 10^3^	6.3 × 10^3^	31 × 10^3^
	10	48	14.1 × 10^3^	4.149	6.77 × 10^3^	4.9 × 10^3^	27 × 10^3^
	15	48	11.08 × 10^3^	4.044	6.18 × 10^3^	4.6 × 10^3^	22 × 10^3^
	20	48	9.16 × 10^3^	3.961	5.59 × 10^3^	3.1 × 10^3^	21 × 10^3^
Pseudomonas (MPN/100 ml)	0	48	94.23	1.974	49.656	26	240
	4	48	80.02	1.903	38.793	23	180
	7	48	71.13	1.852	34.742	22	170
	10	48	60.23	1.779	29.826	21	140
	15	48	43.29	1.636	31.403	17	130
	20	48	34.83	1.541	27.864	14	110
HPC (CFU/ml)	0	48	166.2 × 10^3^	5.220	51.39 × 10^3^	83 × 10^3^	35 × 10^3^
	4	48	152.6 × 10^3^	5.183	45.32 × 10^3^	76 × 10^3^	241 × 10^3^
	7	48	142 × 10^3^	5.152	45.48 × 10^3^	65 × 10^3^	232 × 10^3^
	10	48	129.8 × 10^3^	5.113	44.38 × 10^3^	56 × 10^3^	199 × 10^3^
	15	48	95.4 × 10^3^	4.979	47.34 × 10^3^	43 × 10^3^	182 × 10^3^
	20	48	82.4 × 10^3^	4.916	47.62 × 10^3^	26 × 10^3^	172 × 10^3^

**Fig. 8:**
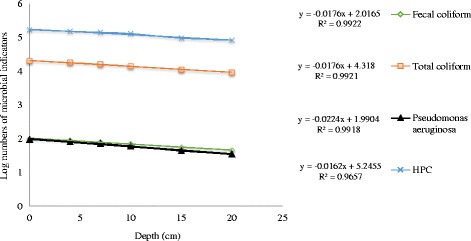
General decay pattern for the microbial indicators in marine sediment

**Fig. 9:**
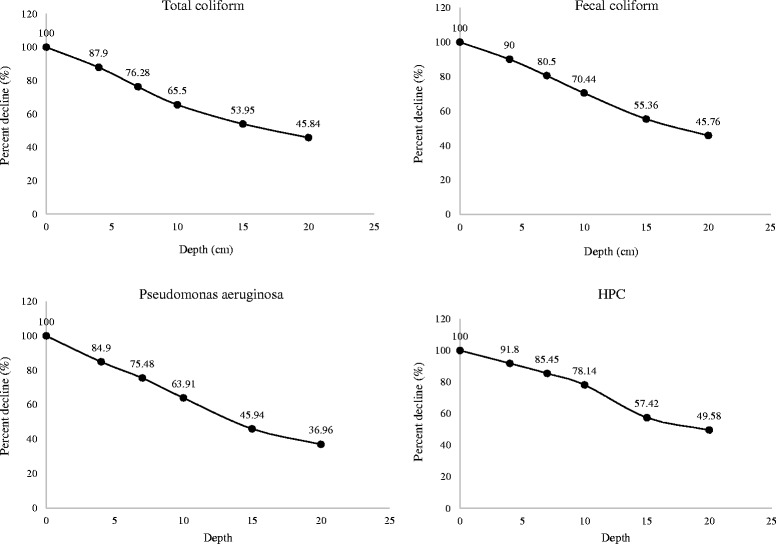
Percent decline (%) of bacteria in marine sediment with depth

As shown in Fig. 8, general decay pattern for examined bacterial indicators in sediments of Bushehr coastal areas are presented. These pattern can be useful to anticipate indicator bacteria numbers in marine environment sediments considering sediment texture and grain size. The highest percent decline of total coliform was found in depth between 0 and 4 cm, but the highest percent decline of fecal coliform, *Pseudomonas aeruginosa* and HPC bacteria were found in depth between 10 and 15 cm (Fig. 9). Haller et al. found that the concentration levels of bacterial indicators decreased with depth. They reported this can be due to decrease in organic matter content, they found out that organic matter content in the sediments samples reduced with depth, from 25% at 0–2 cm to 15% at 10 cm depth [21]. Brinkmeyer et al. detected a considerable reduction in the contents of indicator bacteria from the top 1 cm (10^4^ to 10^5^) to the deeper 15, 30, and 60 cm horizons (10^2^ to 10^3^) [26].

### Grain size analysis

Sediment grain size analysis in Bushehr coastal areas along the Persian Gulf are shown in Table 5. As seen, sediments in five stations (Skele-Jofreh (S2), Gomrok (S4), Skele-Solh Abad (S5), Skele-Jabri (S6), and Shoghab (S8)) have a texture of silt – clay (mud) and a diameter less than 125 μm, but sediments in three stations (TV Park (S1), Daneshjo Park (S3), and Bandargah (S7)), have a texture of fine sand and a diameter between 125-250 μm. Our results revealed that the concentrations of indicators organisms were greater in muddy sediments compare with sandy ones. It is a fact that fine-grained sediment due to the higher surface area to volume ratio has more potential to tend higher concentration levels of bacterial indicators [52–54]. In a study by Lang and Smith, it was concluded that the clay amount of soils is particularly important regarding to abundance of indicator bacteria, as clay particles protected bacteria against predators and higher availability of substrates and moisture than sand particles [55]. A higher level of *E. coli* in sites with higher percent of clay and silt and lower sand has been reported in former studies [56, 57]. Burton et al. found that *E. coli* survival was higher in sediments containing at least 25% clay (particles less than 2 mm in diameter), presumably due to enhanced attachment to the finer sediment particles [58]. In contrast to our study, Cinotto et al. reported that *E. coli* survival was higher in sediments with mainly big particles (ranging in size from 125 to 500 mm), possibly due to bigger sediment particles facilitate increased permeability and accessibility of nutrients [59].

**Table 5:** Sediment grain size analysis in Bushehr port coasts at various stations

Station	Mesh (mm)								Texture
	4	2	1	0.5	0.25	0.125	0.063	<0.063	
1	0.934	3.728	5.756	8.938	35.327	38.158	6.794	0.437	Fine sand
2	0.283	1.412	1.226	3.796	8.443	29.172	53.848	1.820	Silt – clay
3	1.110	4.084	4.809	10.844	16.576	42.916	14.540	5.120	Fine sand
4	6.892	3.325	2.697	2.196	2.777	18.742	58.756	4.613	Silt – clay
5	1.418	1.141	0.710	0.411	0.729	31.861	64.316	0.740	Silt – clay
6	0.568	0.561	1.02	1.694	4.372	21.813	67.44	2.525	Silt – clay
7	0.221	0.132	0.676	10.328	17.910	37.431	30.254	3.046	Fine sand
8	0	0.242	0.418	0.951	4.716	39.661	51.831	2.18	Silt – clay

## Conclusion

The present study was the first attempt to survey indicators bacteria profiles in seawater and sediment of coastal area along the Persian Gulf. Our study revealed that there was a reverse correlation between the levels of indicator bacteria and salinity of seawater samples. The levels of indicator organisms were 10 to 100 times higher in sediments than in seawater samples. The concentration levels of indicators bacteria were higher in muddy sediments compare with sandy ones. Our results revealed that the concentration levels of bacterial indicators decreased with depth. Our presented models in this study can be useful models to anticipate indicator bacteria numbers in marine environment sediments considering sediment texture and grain size. The orders of indicator bacteria numbers in seawater and sediment samples were HPC *>* total coliform *>* fecal coliform *> pseudomonas aeruginosa*. The concentration levels of indicator bacteria in sampling stations showed that coastal areas along the Persian Gulf are facing a wide variety of anthropogenic sources. Finally monitoring and mitigation measures of marine environment particularly in places with entertainments uses are greatly suggested.

## Abbreviations

GIS: Geographical Information System
HPC: Heterotrophic Plate Count
